# Enhancement of reactive oxygen species production in triple negative breast cancer cells treated with electric pulses and resveratrol

**DOI:** 10.37349/etat.2023.00122

**Published:** 2023-02-28

**Authors:** Pragatheiswar Giri, Ignacio G. Camarillo, Raji Sundararajan

**Affiliations:** 1 School of Engineering Technology, Purdue University, West Lafayette, IN 47907, USA; 2 Department of Biological Sciences, Purdue University, West Lafayette, IN 47907, USA; 3 Purdue University Center for Cancer Research, West Lafayette, IN 47907, USA; NGO Praeventio, Estonia

**Keywords:** Resveratrol, triple negative breast cancer, reactive oxygen species, MDA-MB-231 cells, electrical pulses

## Abstract

**Aim:**

Triple negative breast cancer (TNBC) is difficult to treat since it lacks all the three most commonly targeted hormone receptors. Patients afflicted with TNBC are treated with platinum core chemotherapeutics, such as cisplatin. Despite the initial effective anticancer effects of cisplatin, TNBC attenuates its effect and develops resistance eventually, which results in tumor reoccurrence. Hence, there is a critical demand for effective, alternative, and natural ways to treat TNBC. Towards this, a promising technique for inhibiting TNBC cell proliferation involves promoting the production of reactive oxygen species (ROS), which triggers pro-apoptotic caspases 9 and 3. Resveratrol (RESV), an active bio compound found in naturally available fruits, such as grapes, is utilized in this research for that. In addition, electrochemotherapy (ECT), which involves the application of electrical pulses (EP), was utilized to enhance the uptake of RESV.

**Methods:**

MDA-MB-231, human TNBC cells were treated with/out RESV, and eight 600–1,000 V/cm, 100 μs pulses at 1 Hz. The cells were characterized by using various assays, including viability assay, and ROS assay.

**Results:**

A TNBC cell viability of as low as 20% was obtained at 24 h (it was 13% at 60 h), demonstrating the potential of this novel treatment. ROS production was the highest in the combination of EP at 1,000 V/cm along with RESV at 100 μmol/L.

**Conclusions:**

Results indicate that RESV has the potential as an anti-TNBC agent and that EP + RESV can significantly enhance the cell death to reduce MDA-MB-231 cell viability by increasing ROS production and triggering apoptosis.

## Introduction

Reactive oxygen species (ROS) are a family of highly reactive molecules that are evolutionarily conserved since bacteria and *Escherichia coli* (*E. coli*) [1, 2]. It might also be thought of as the first messenger directly involved in regulating the activity of a transcription factor. ROS are produced in mitochondria, peroxisomes, and other organelles [3]. ROS are also produced in response to physical agents, such as ultraviolet rays, heat, and light, as well as after chemotherapy and radiation therapy in cancer [3], leading to cell death by apoptosis. The action of ROS is found to be a double-edged sword; at lower levels, ROS increase cell proliferation [4], but at higher levels, cause cell death as reported in several studies [4, 5]. Increased ROS-induced apoptosis has been reported in cancer cells, due to chemotherapy or radiation therapy, indicating the potential role of ROS modulation in anticancer combinational therapies [5–7]. It is found that photodynamic therapy is more suitable, which is based on the generation of ROS after stimulation by light. A partial list of various ROS-inducing drugs is shown in Table 1 [3].

**Table 1.** A partial list of anticancer drugs based on increased ROS production

**Drug (name)**	**Cancer treated**	**Mechanism to increase ROS**	**References**
Platinum drugs	Breast cancer (in combination with PARP inhibitors)	ROS-dependent DNA damage	[8, 9]
Imatinib	Melanoma	Loss of mitochondrial membrane potential	[10]
Doxorubicin	Kaposi’s sarcoma, breast, and bladder cancer	Fenton’s reaction and electron leakage	[11]
5-Fluorouracil	Colon and rectal cancer	P53 dependent ROS	[12, 13]

PARP: poly (ADP-ribose) polymerase

In this research, the combined effects of electrical pulses (EP) + resveratrol (RESV) on the triggering of ROS in MDA-MB-231, human triple negative breast cancer (TNBC) cells were studied. TNBC cells were chosen because there is an unmet need for the treatment of TNBC. TNBC lacks all the three most commonly targeted hormone receptors, namely, the estrogen receptor (ER), progesterone receptor (PR), and human epidermal growth factor receptor 2 (HER2) amplification [14], and hence there are no targeted therapies and is also associated with poor prognosis. The 5-year survival rate of TNBC patients is 30% compared to 66% for other breast cancer phenotypes [15, 16]. Conventional chemotherapies also have severe side effects, in addition to being costly. In addition, TNBC cells have distinct metabolic characteristics to support high rates of proliferation [17]. Phytochemicals have been found to modulate multiple cellular signaling pathways while causing no or little toxicity to normal cells. There is a growing interest in the use of natural compounds, such as RESV for various cancers, including breast cancer [18].

RESV, a natural polyphenol was chosen due to its antioxidant, anti-cancerous, and other characteristics [18], along with its mild and gentle effect on bodies. RESV acts in several ways: antiproliferation, induction of apoptosis, epigenetic response, epithelial-mesenchymal transition (EMT)/metastasis reduction, sensitization to chemotherapy, and others [19].

RESV (3,5,4’-trihydroxy-trans-stilbene) is a member of the stilbenoids subclass of polyphenols [18]. This natural polyphenol has been found in over 70 plant species; out of those, most potently in grapes [20]. The isolation of RESV is from plant species, called *Cassia quinquangulata*, based on the inhibition of the cyclooxygenase-1 (COX-1) [20].

Multiple studies reported that RESV has a very high antioxidant potential as a natural food ingredient [21, 22]. The topical application of RESV reduced pedal oedema in a rat model of carrageenan-induced paw inflammation and decreased tumorigenesis in a mouse skin cancer model, which supports RESV as an effective anticancer compound [23, 24]. RESV-mediated antioxidant defence is via a nuclear factor E2-related factor 2 (Nrf2)-dependent signalling pathway, suggesting that RESV plays important roles in the regulation of cellular antioxidant responses to breast cancer [25]. RESV has been shown in animal studies to be well tolerated with little toxicity. In rats, doses as high as 3,000 mg RESV/kg body weight per day for four weeks were found to have no adverse effects [26]. RESV also influenced the activity of the B cell lymphoma-2 (Bcl-2) regulator nuclear factor-B (NF-B) [27]. Electrophoretic mobility shift assay (EMSA) experiments revealed a significant decrease in the binding of P65 to DNA at reticuloendothelial system (RES) concentrations that induce apoptosis [27]. The decrease in NF-B activity could be attributed to a lower level of nuclear P65, which was most likely caused by an increase in cytosolic inhibitor-kappa B (I-kB), which retained NF-B in the cytosol, as demonstrated by immunoprecipitation experiments [28].

To enhance the uptake of RESV, EP was used to transiently open pores in the plasma cell membranes, which are usually nonpermeable or less permeable. This technique is known as electroporation [29–32].

The combination of electroporation and drug is known as electrochemotherapy (ECT) [33–36]. ECT can target multiple factors and is a clinically tested and proven technique for treating advanced, inoperable cancers [37]. ECT creates a temporary permeability by using EP to enhance the drug to target cancer cells effectively against TNBC. ECT-based drug enhancement proceeds without causing severe toxicity or drug resistance, because it does not rely on receptors or hormones that target cancer cells [38].

## Materials and methods

### Cell lines

MDA-MD-231, an epithelial, human TNBC cell line, first deduced from a 51-year-old Caucasian female breast cancer patient was used. The cells were incubated and cultured in Dulbecco’s modified eagle medium (DMEM; 11965084, Thermo Fisher, USA) containing 10% fetal bovine serum (FBS; A5256801, Thermo Fisher, USA) along with 1% penicillin-streptomycin (PS; 15070063, Thermo Fisher, USA) at suitable humidity and optimal growth conditions.

### RESV drug

**Figure 1.**
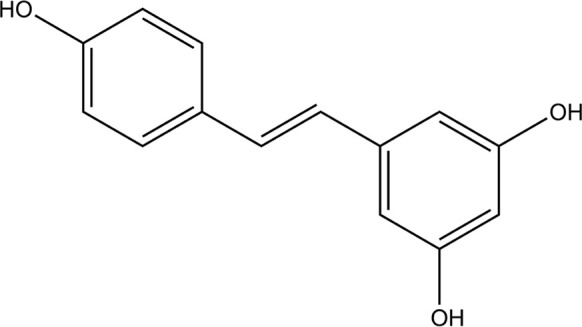
Chemical structure of RESV

RESV stock solution (15 mmol/L, Sigma-Aldrich, USA) was prepared using dimethyl sulfoxide (DMSO; J66650, Thermo Fisher, USA). The stock was diluted to obtain 10–150 μmol/L RESV treatment concentrations. The chemical structure of RESV is shown in Figure 1. Short-term, low doses of RESV do not appear to have any side effects (at 1.0 g) [39]. Otherwise, side effects such as nausea, vomiting, diarrhea, and liver dysfunction may occur at doses of 2.5 g or higher per day in patients with non-alcoholic fatty liver disease.

### EP application

A BTX ECM 830 square wave pulse generator (76271, Genetronics Inc. San Diego, CA, USA) was used to apply eight unipolar, square wave pulses of 600–1,000 V/cm of 100 μs long, at 1 s intervals. The MDA-MB-231 cells were collected for electroporation at a concentration of 1 × 10^6^ cells/mL and suspended at 600 μL per cuvette, with a 4 mm gap between the cuvette walls. Treated samples were dispensed to either 96-well plates or petri dishes, depending upon the assay type.

### MT real-time viability assay

**Figure 2.**
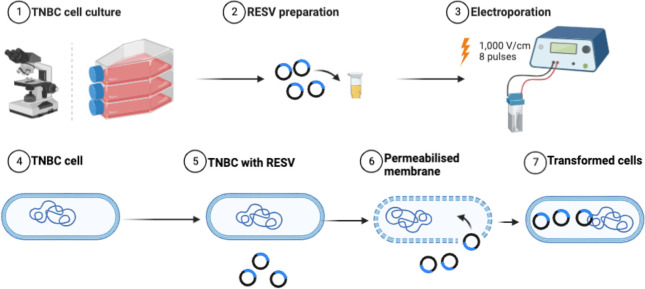
Experimental workflow of combinational studies illustrating the enhancement of RESV delivery induced into the TNBC cells

The treated samples, including control (no treatment), RESV only, EP only, and EP + RESV, at 1.0 × 10^6^ cells/mL concentration were seeded into 96-well plates, per Promega’s protocol. The cells were supplied with 55 μL of prepared media in each well. They are then incubated with optimal growth conditions for 24–60 h. To study the cell viability, Real-Time-Glo^TM^ MT Cell Viability Assay (G9711, Promega, USA) was used. Synergy LX Multi-Mode Reader (SLXATS, BioTek Instruments, USA) was utilized for recording the luminescence (Lum). Equation 1 was used to calculate the percentage of cell viability. The experimental workflow is shown in Figure 2.

Equation 1:

$$Cell\;viability\;(\%)=\frac{Treated\;cell\;Lum\;value}{Control\;Lum}\times 100\%$$

### ROS assay

The measurement of ROS was also performed according to the manufacturer protocol. First, 20 μL (20,000 cells) of treatment samples were added to a 96-well plate, with an additional 60 μL of cell media. After 18 h incubation, 20 μL of hydrogen peroxide (H_2_O_2_; G882B, Promega, USA) substrate from ROS-Glo^TM^ (G8821, Promega, USA) was added and further incubated for 6 h. The luciferin precursor was formed by the direct reaction between the H_2_O_2_ present in the media and the H_2_O_2_ substrate added externally from the kit. Subsequently, a 100 μL luciferase detection reagent is introduced to each well, and relative Lum units (RLU) measurement using Synergy HTX multi-mode microplate reader (SLXATS, BioTek Instruments, USA) is taken after 20 min of incubation time. During that period, the luciferin precursor is converted to luciferin in the presence of the ROS-Glo^TM^ substrate detection solution. The H_2_O_2_ in the sample is proportional to the light signal. ROS levels were measured at 12, 24, and 48 h to investigate their change with time.

### Statistical analysis

All experiments were done in triplicates. To establish the significance between the different treatment data, analysis of variance (ANOVA) was utilized [40]. The level of confidence was set to 95% along with constant variance, and data following a normal curve was confirmed prior to the ANOVA tests.

Data from assays were also subjected to Tukey’s comparison test [41] to establish normality and homoscedasticity on TNBC cell treatments and activities. Based on the critical value from the Tukey significance test, samples are assigned letter grades, where the same letter(s) means no statistical difference, and different letter means that they are statistically different. Brown-Forsythe test was also performed along with ANOVA which provides more robustness to the significance.

## Results

### RESV only and EP only studies

**Figure 3.**
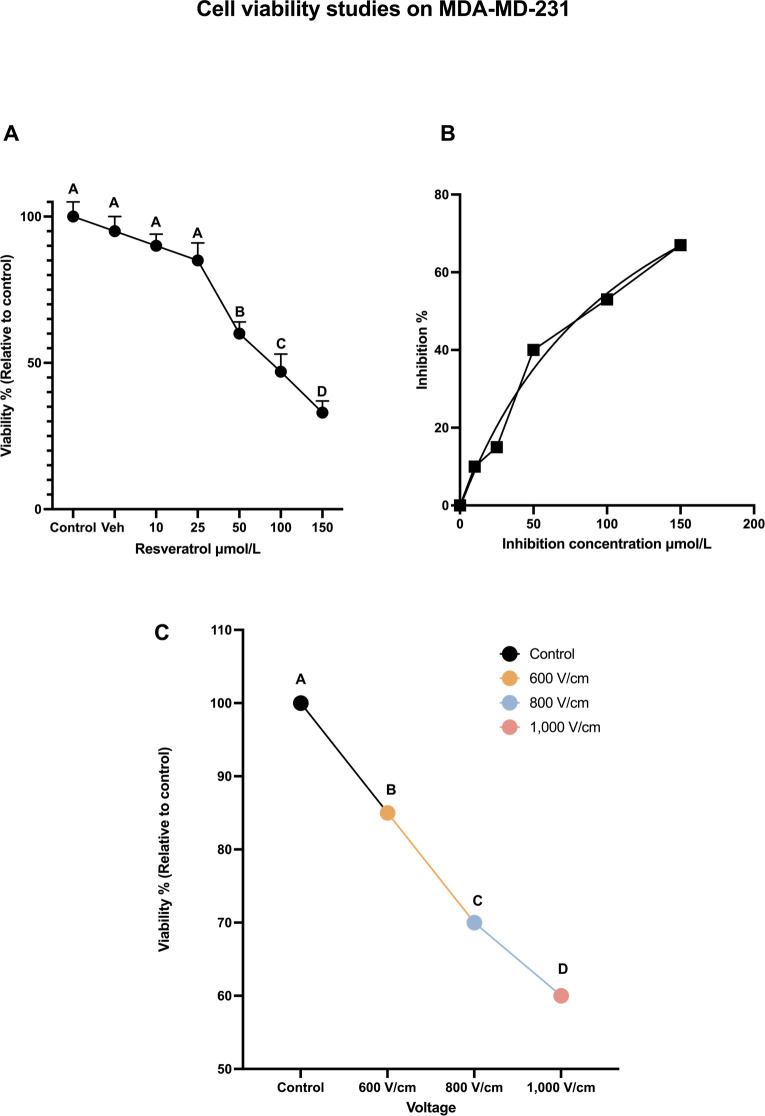
Cell viability studies on MDA-MD-231. (A) The dose-dependent cell viability of MDA-MB-231 cells with various concentrations of RESV; (B) inhibition concentration curve indicating cell death due to RESV only at various concentrations; (C) the percentage of cell viability with respect to control when various voltages are administered. The same letter A for samples of control, and up to 25 μmol/L, indicates that there are no statistically significant changes. The different letters, B, C, and D indicate that the viabilities of these samples are significantly different. Veh: vehicle

The variation of cell viabilities (dose curve), relative to control (normalized to 100%), at 24 h, along with Tukey letters grade are shown in Figure 3A. The dose curve indicates that RESV causes a dose-dependent reduction of MDA-MB-231 TNBC cell viability. There is a cell viability drop of only 10% at 10 μmol/L and 15% at 25 μmol/L (compared to the control). However, the viability dropped to 60% at 50 μmol/L, to 50% at 100 μmol/L, and to 45% at 150 μmol/L, indicating the effect of higher concentrations on the cell viability.

Inhibition concentration [42] results show that RESV alone caused significant cell death in a dose-dependent manner with the half-maximal inhibitory concentration (IC_50_) of 35.78 μmol/L after 24 h shown in Figure 3B.

The effect of EP only is shown in Figure 3C, which was observed 24 h after treatment. As the strength of the electric field increases, cell viability decreases. After 24 h, the viability at 800 V/cm was 67% compared to the control (at 100%). The viability dropped to 54% at 1,000 V/cm.

Tukey test results indicate the statistical significance of these results with letters A, B, C, and D indicating that the viabilities of these samples are significantly different.

### EP with RESV combination studies

Electroporation at various voltages only or RESV, up to 50 μmol/L dose alone did not produce a notable decrease (< 50%) in viability as shown in Figures 3A and 3C. Hence, further experiments were done with the combination of EP and RESV.

**Figure 4.**
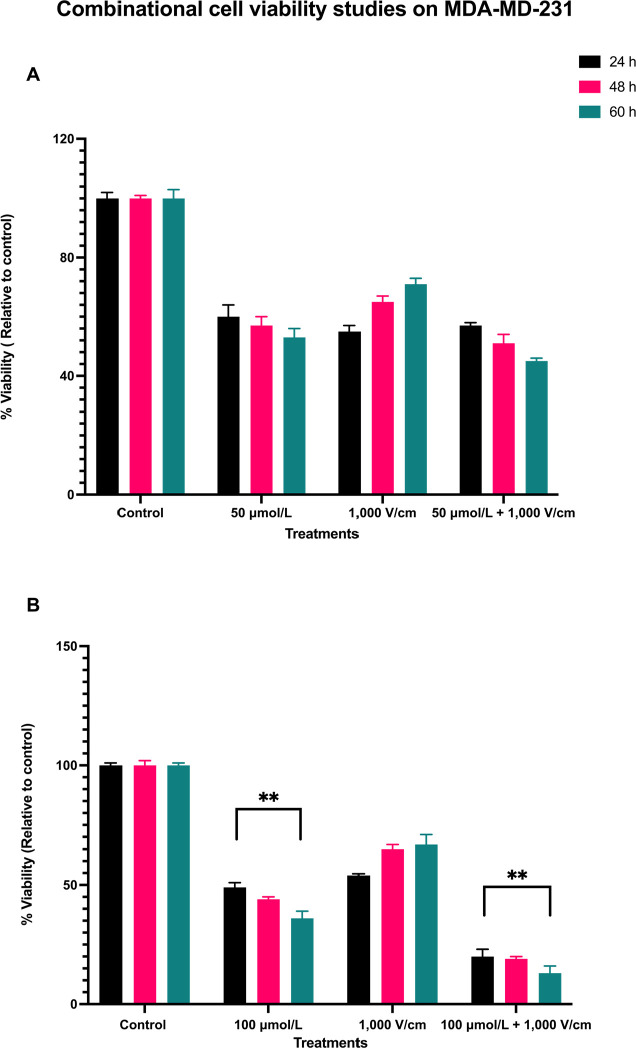
Combinational cell viability studies on MDA-MD-231. (A) Viability of MDA-MB-231 cells with 50 μmol/L RESV along with 1,000 V/cm compared to control and drug only treatments; (B) viability of MDA-MB-231 cells at 100 μmol/L + 1,000 V/cm compared with drug only and control. ^**^ indicates the significant differences (*P* < 0.0001)

The results for a combination of 1,000 V/cm and 50 μmol/L at 24, 48, and 60 h are shown in Figure 4A. There is still not much cell death with this combination. This indicates that not only RESV alone at 50 μmol/L is less effective in killing the cells, but also when combined with 1,000 V/cm. Statistical analysis by ANOVA indicated a *P* value of less than 0.4109 (Table 2), which shows no significant differences between these viabilities. Hence, the experiments were with a combination of 100 μmol/L and 1,000 V/cm.

**Table 2.** Summary of ANOVA analysis on MDA-MB-231 cell line to assess the metabolism (MT) cell viability assay with all the treatments with low doses and voltages

**ANOVA table**	**Sum-of-squares (SS)**	**Degree of freedom (DF)**	**Mean square (MS)**	**F**	***P* value**
Treatment	991	10	99	0.9	*P* < 0.4109
Residual	204	36	5.56	-	-
Total	1,195	46	-	-	-

-: blank cell

The synergistic combinational effect of 100 μmol/L and 1,000 V/cm at 24, 48, and 60 h timepoints are displayed in Figure 4B. There is a notable decrease in cell viability; it was as low as 20% (control, normalized to 100%) at 24 h, 19% at 48 h, and 13% at 60 h.

The viability of MDA-MB-231 cells was reduced at 24 h initially for all the samples, but they eventually increased for EP only, at 48 and 60 h, indicating that EP alone does not cause cell death over a prolonged period. With a combination of 100 μmol/L and 1,000 V/cm, there was a consistent time-dependent decrease in cell viability, even after 24 h, unlike those at EP only. The combination, EP + RESV, produced a synergetic effect on MDA-MB-231 cells. The *P* < 0.0001 (Table 3) indicates that these results are statistically significantly different, which are indicated by ^**^ respectively.

**Table 3.** Summary of ANOVA analysis on MDA-MB-231 cell line to assess the MT cell viability assay with all the treatments

**ANOVA table**	**Sum-of-squares (SS)**	**Degree of freedom (DF)**	**Mean square (MS)**	**F**	***P* value**
Treatment	583.5	3	97.25	19.5	^**^
Residual	119.7	24	4.984	-	-
Total	703.2	27	-	-	-

-: blank cell; ^**^ *P* < 0.0001

**Figure 5.**
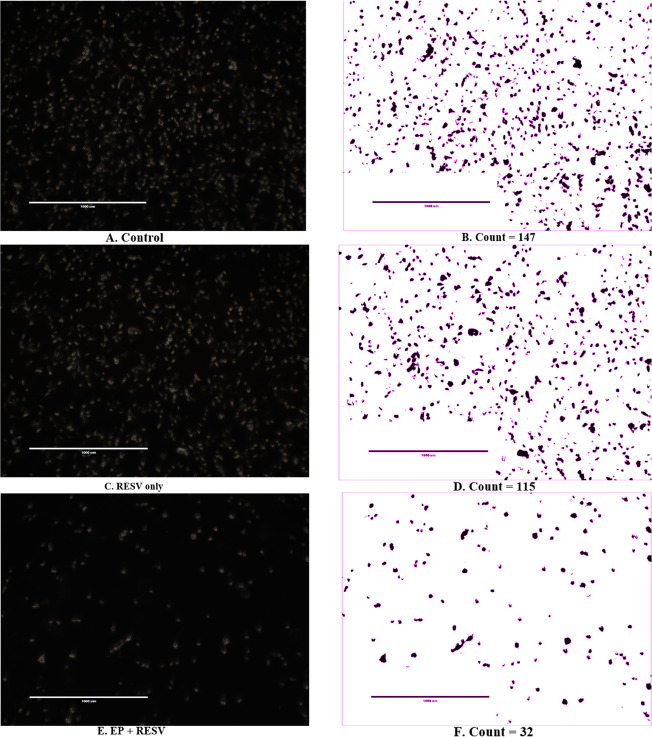
MDA-MB-231 live cell imaging and image segmentation count at 24 h after treatments (400×)

The live cell images of MDA-MB-231 at 24 h after treatments for control, RESV only, and EP + RESV, and the corresponding Matrix Laboratory (MATLAB) computed quantitative values are shown in Figure 5. The control (Figure 5A) has the maximum number of cells among all the treatments. The RESV only depicts more confluency (Figure 5C), and a smaller decrease in cell numbers compared to the control (115/147), while the EP + RESV has the least number of cells (32/147), correlating with the viability values. The varied morphological changes between these cell samples were also observed. While the control cells were more circular and well-defined, they were distorted for RESV only samples. The cells were maximumly distorted for EP + RESV (Figure 5E), indicating that they were undergoing distress. The corresponding MATLAB counts are shown in Figures 5B, 5D, and 5F.

### ROS assessments

**Figure 6.**
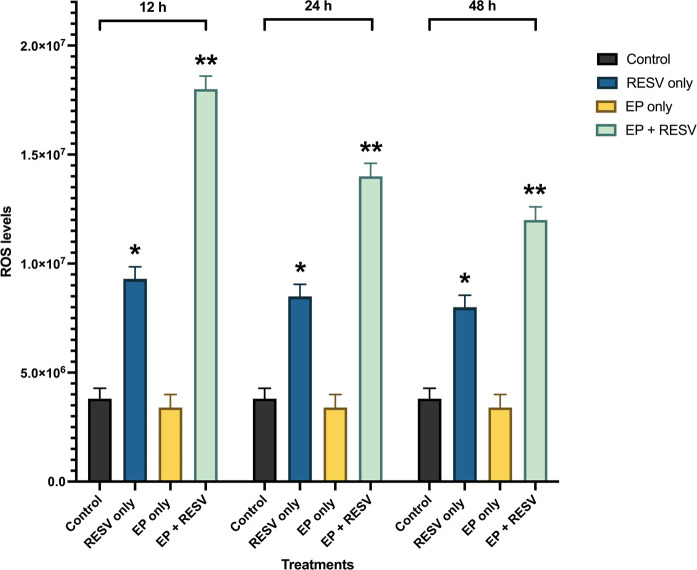
ROS levels for various treatments in MDA-MB-231 at 12, 24, and 48 h time points. ^*^, ^**^ indicates the significant differences (*P* < 0.0001)

The quantitative intracellular ROS levels at multiple time points of 12, 24, and 48 h are shown in Figure 6. The ROS levels are noticed to be the highest in 12 h compared to the 24 h and 48 h time points. At 12 h, EP at 1,000 V/cm along with RESV at 100 μmol/L produced the maximum ROS production. At 24 h, it was over 3.68x control, and at 48 h, it was 3.3x, compared to control-indicating the strong impact of EP + RESV on the ROS production, at all-time points.

It was also observed that with EP only treatment, the ROS levels were not significantly different compared to the control at all the time points, which indicates that the EP + RESV treatments are synergetic in nature and not additive.

Also, RESV only treatment produced lower than EP + RESV combination at all the time points. EP only treatment produced the lowest, almost at the same level as the control. Compared to RESV only and EP only treatments, EP + RESV treatment increased ROS production the most. This causes more oxidative stress in the MDA-MB-231 cells, leading to apoptosis [28–31], leading to cell death as illustrated by the viability values.

The initial high levels of ROS also correlate to the number of tumor cells present. Comparing the ROS levels to the viability levels, it is noticed that as the viability decreases, there are significantly fewer viable cells and a reduction in ROS levels. In all the time points 12, 24, and 48 h, the combination of EP + RESV generated the highest levels of intracellular ROS levels in the MDA-MB-231 cells.

ANOVA analysis (Table 4) shows *P* < 0.0001, indicating that the treatments are significantly different from each other. The significant differences between the samples are indicated by ^**^.

**Table 4.** Summary of ANOVA analysis of the ROS levels of the MDA-MB-231 cells

**ANOVA table**	**Sum-of-squares (SS)**	**Degree of freedom (DF)**	**Mean square (MS)**	**F**	***P* value**
Treatment	4,168	3	138	443.5	^**^
Residual	2,505	8	3,132	-	-
Total	6,673	11	-	-	-

-: blank cell; ^**^ *P* < 0.0001

## Discussion

ROS is vital for apoptotic cell death and the production of ROS levels due to EP + RESV were investigated in this research. Prior studies using other drug compounds have also revealed increases in ROS levels when treated with electric pulses leading to cell death via apoptosis [43–45].

Treatment with natural compounds, such as RESV and anticancer drugs causes an imbalance in the reactive oxygen levels, which leads to apoptosis via P53 and mitochondrial cytochrome release [46]. This imbalance between the production and elimination of ROS, and the biological system’s capacity to quickly detoxify the reactive intermediates or to repair the damage that results is known as oxidative stress [47]. ROS production can cause oxidative stress with severe DNA damage to the cells via the initiation, promotion, and progression mechanisms [48]. The production of ROS was seen to be linked to DNA strands, tumor suppressors, and thereby promoter silencing of multiple genes [49].

Evidence indicates that the production of intracellular ROS is critical for triggering apoptosis. In addition, cancer cells produce more ROS than normal cells and increased ROS induces apoptosis in cancer cells [50]. Increased ROS levels in cancer cells are linked to the activation of P53, a key tumor suppressor. P53 is essential for cell cycle regulation, DNA damage, cell apoptosis, and tumor suppression. ROS is also important in the regulation of proapoptotic Bcl-2-associated X protein (Bax) and anti-apoptotic Bcl-2. The upregulation of Bax and the downregulation of Bcl-2, along with the higher expression of P53 resulting in apoptosis is reported [51]. ROS also stimulates the mitogen-activated protein kinase (MAPK) pathway. The MAPK superfamily is made up of protein kinases, such as extracellular signal-regulated kinase (ERK), c-Jun N-terminal kinase (JNK), and P38. MAPK is activated in response to cellular stress and metabolism. When exposed to oxidative stress, ERK promotes cell survival, whereas P38 and JNK activation cause apoptosis [52].

Mitochondria have been identified as one of the most important target organelles during ROS-mediated cancer cell apoptosis [44, 53]. Intracellular ROS and subsequent Bax/Bcl-2 modulation are preceded by mitochondrial membrane potential collapse, which has been linked to the initiation of a caspase cascade leading to cell death [54]. RESV’s antioxidant properties, activated by ROS production include the regulation of numerous signaling pathways involved in the carcinogenesis process [55]. This compound inhibits many kinases, including protein kinase C (PKC), MAPK, and inhibitor of kB kinase (IKK), as well as some transcription factors activated by ROS, including NF-kB, signal transducer and activator of transcription 3 (STAT3), hypoxia-inducible factor 1 (HIF-1), and activator protein-1 (AP-1) [56].

RESV was also seen to inhibit lactate production significantly. Studies indicate that RESV significantly suppresses glucose metabolism in cancer cells. Additionally, RESV was seen to reduce glucose uptake by decreasing glycolytic metabolism, as evidenced by decreased lactate production. Among the major determinants of tumor glycolytic flux, reduced glucose transporter protein 1 (Glut-1) expression was found to be more important than hexokinase activity [57–59].

The release of extracellular receptor calcium, which is reflected in a swift rise in intracellular calcium was observed [60]. This occurrence of RESV activating the inositol triphosphate (IP_3_) receptor (IP_3_R) with diacylglycerol (DAG) through either an extracellular receptor or an intracellular target is followed by the subsequent calcium uptake by the mitochondria, the release of cytochrome c, and an increase in ROS production [61]. Mitochondrial calcium-induced calcium-release (mCICR) activates calpain, which then cleaves various substrates, such as calcium exchangers and pumps, such as plasma membrane calcium + transporter plasma membrane calcium ATPase 1 (PMCA1), causing an increase in intracellular calcium and thus stimulating cell death [62].

**Figure 7.**
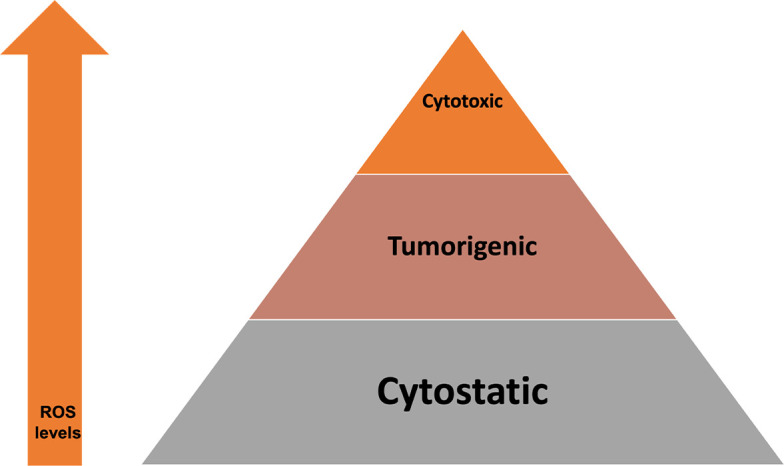
Impact of intracellular ROS levels on cell death

As mentioned before [5], at low transient levels, ROS can lead to cellular proliferation and survival signaling pathways, while at higher levels lead to cell death via apoptosis. The impact of intracellular ROS at various levels are illustrated in Figure 7. This correlates well with our results, where with RESV only and EP only, there were less ROS production and less cell death, compared to EP + RESV, with the highest ROS production and the highest cell death.

In another study, the intracellular ROS production was seen to be higher with RESV, which resulted in apoptosis in non-metastatic, ER positive MCF-7, human breast cancer cells [63]. It was observed that higher doses of RESV were needed to inhibit cell proliferation and induce apoptosis in TNBC [64].

**Figure 8.**
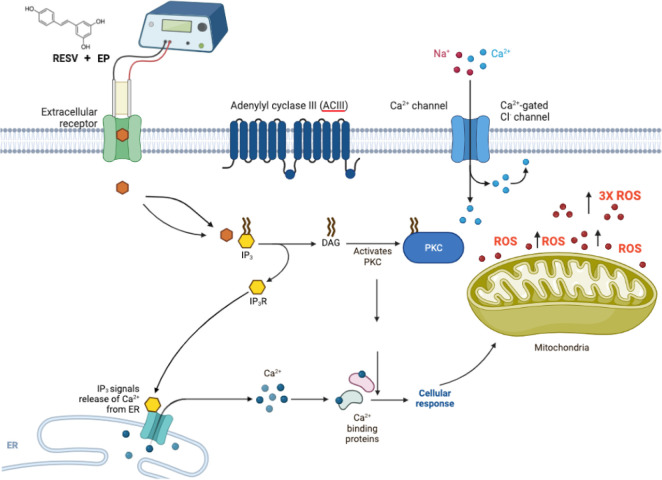
Suggested possible mechanism of action of RESV shown with putative binding mode of RESV, combined with EP for delivery enhancement, that induces apoptosis

The mechanism of action of RESV, with a putative binding mode of RESV, inducing apoptosis, with EP enhancing RESV uptake is shown in Figure 8. Further studies are required to understand the mechanism and impact of ROS levels on TNBC cells, due to EP + RESV.

To conclude, the results indicate that RESV has the potential as an anti-TNBC agent and that EP + RESV can significantly enhance cell death and reduce MDA-MB-231 cell viability. This could be due to the triggering of ROS at higher levels, leading to cell death via apoptosis. The morphological changes in the cell images corroborate this. In general, there were no substantial changes in viability or ROS levels when TNBC cells were treated with either RESV or EP alone, indicating the potential of this novel combination treatment for possible future clinical applications.

## Abbreviations

ANOVA: analysis of variance
Bax: B-cell lymphoma-2-associated X protein
Bcl-2: B cell lymphoma-2
ECT: electrochemotherapy
EP: electrical pulses
H_2_O_2_: hydrogen peroxide
IP_3_: inositol triphosphate
MAPK: mitogen-activated protein kinase
NF-B: nuclear factor-B
RESV: resveratrol
ROS: reactive oxygen species
TNBC: triple negative breast cancer

## Declarations

### Copyright

© The Author(s) 2023.
